# Perspectives of a scrapie resistance breeding scheme targeting *Q211*, *S146* and *K222* caprine *PRNP* alleles in Greek goats

**DOI:** 10.1186/1297-9716-45-43

**Published:** 2014-04-09

**Authors:** Eirini Kanata, Cynthia Humphreys-Panagiotidis, Nektarios D Giadinis, Nikolaos Papaioannou, Minas Arsenakis, Theodoros Sklaviadis

**Affiliations:** 1 Department of Genetics, Development and Molecular Biology, School of Biology, Aristotle University of Thessaloniki, Thessaloniki, Greece; 2 Department of Pharmaceutical Sciences, School of Health Sciences, Aristotle University of Thessaloniki, Thessaloniki, Greece; 3 Faculty of Veterinary Medicine, Farm Animal Clinic, Aristotle University of Thessaloniki, Thessaloniki, Greece; 4 Laboratory of Pathology, Faculty of Veterinary Medicine, Aristotle University of Thessaloniki, Thessaloniki, Greece

## Abstract

The present study investigates the potential use of the scrapie-protective *Q211 S146* and *K222* caprine *PRNP* alleles as targets for selective breeding in Greek goats. Genotyping data from a high number of healthy goats with special emphasis on bucks, revealed high frequencies of these alleles, while the estimated probabilities of disease occurrence in animals carrying these alleles were low, suggesting that they can be used for selection. Greek goats represent one of the largest populations in Europe. Thus, the considerations presented here are an example of the expected effect of such a scheme on scrapie occurrence and on stakeholders.

## Introduction, methods and results

Scrapie is an extensively studied, infectious, fatal neurodegenerative disorder of sheep and goats. While the disease has been known since the 18^th^ century, only in relatively recent years have we been able to characterize it at the molecular level as a conformational conversion of the normal α-helix-rich PrP^C^ protein to its abnormal β-sheet rich isoform, designated PrP^Sc^. According to the protein-only hypothesis, PrP^Sc^ is the infectious agent. Single nucleotide polymorphisms (SNP) in the *PRNP* gene, encoding the PrP^C^ protein, have been strongly associated with scrapie in sheep and the relevant information has been successfully applied in European scrapie-eradication programs for sheep.

Unfortunately, the same associations between ovine *PRNP* polymorphisms and scrapie resistance cannot be directly applied to the case of scrapie in goats, since the study of caprine *PRNP* gene variability and the detection of polymorphisms associated with protection against the disease have only recently gained increasing attention. The caprine *PRNP* gene is highly polymorphic [1] and several polymorphisms, including *I142M*, *H143R*, *R154H*, *N146S/D*, *R211Q*, and *Q222K,* have been linked with protection against scrapie in field studies [2-7]. Although none of these has been shown to confer complete resistance in heterozygous animals, the *S146, K222* and *Q211* alleles have been associated with the highest degree of protection in experimentally challenged goats and in transgenic mouse lines [8-11]. While the exact mechanism through which these variants confer resistance has not yet been determined, it is believed that, as in the case of sheep [12,13], PrP^C^ variants with the aforementioned polymorphisms are less prone to conversion upon interaction with PrP^Sc^[14].

The Greek goat population is estimated to almost 5 million, one of the largest in Europe. Goat farming is a highly dynamic agricultural sector in Greece, contributing by almost 18% to the total agricultural income. Greek goats are dairy animals, also bred for meat production. They belong mainly (70%) to the indigenous Greek goat breed (*Capra prisca*), and to other high- and medium-milk yielding dairy breeds (Alpine, Saanen, Damascus), which were imported (1920-1950), aiming at production trait improvement. Due to subsequent uncontrolled crosses between the indigenous and the above mentioned breeds, the current Greek goat population is not homogenous, and the degree of each breed contribution is not well defined. This is observed not only in Greece, but in other countries as well. Since 1986 when the first cases of scrapie were reported in an affected flock of Greek sheep, the disease has occurred in both sheep and goats. Scrapie prevalence in Greek goats was estimated to 47.1%, for 2011. According to the current Greek legislation (EK4922/131349), holdings where scrapie cases have been detected in either sheep or goats are subject to complete destruction, provided that more than 5% of the animals display clinical signs. Alternatively, animals can be set under strict surveillance, provided that all sheep carrying susceptible genotypes, and goats older than three months are sacrificed and incinerated. While the genotyping of all sheep for *PRNP* codons 136, 154 and 171 is performed, only rapid tests on tissue samples are applied to the goats of these holdings. Moreover, systematic *PRNP* genotyping of Greek goats is not foreseen.

In order to control the disease spread, improve animal welfare, and reduce economic losses due to animal sacrifice, the implementation of a breeding program for increasing scrapie resistance in goat populations would be desirable as well. The planning and implementation of such a program require thorough scrapie-strain typing and detection of caprine scrapie-resistance alleles. Furthermore, its application to a given population presupposes that both the caprine *PRNP* gene variability and the frequencies of resistance-associated alleles in the population are known.

To date, very few studies on *PRNP* variability in Greek goats have been published [3,15,16]. Most of these [3,15] focused on single scrapie-affected holdings, and thus, an estimation of the overall *PRNP* variability in the Greek goat population has not been assessed. The aim of the present study was to (a) determine the degree of *PRNP* variability in a high number of healthy Greek goats, with special emphasis on bucks, and (b) to interpret the results, discussing the applicability of possible selective breeding programs for the control, and eradication of goat-scrapie.

Samples corresponding to 436 clinically healthy goats, from 39 holdings across 7 prefectures of northern Greece were analyzed. Interestingly, a great number of the tested samples originated from holdings located within a scrapie-endemic region. Between 5 and 25% animals from each herd were sampled, with this sampling mainly targeting males. All the available bucks from 59% of the tested flocks were included in the study.

Blood samples were collected on Whatman FTA Classic blood cards (Cat No WB12 0205) and genomic DNA was extracted using a manual methanol-precipitation method [17]. The full ORF of the caprine *PRNP* gene was amplified using the AmplitaqGold PCR Master Mix (Invitrogen, Cat No 4318739), with the Pr3F (5’ tgg gca tat gat gct gac ac 3’), and Pr3R (5’ aaa cag gaa ggt tgc ccc ta 3’) primers. PCR reactions were performed using 0.24 μM of each primer and 1 μg of the genomic DNA preparation. Thermal cycling included: (a) an initial denaturation step at 95 °C for 15 min, (b) 41 cycles of denaturation at 94 °C for 30 s, followed by primer annealing at 59 °C for 45 s, and primer extension at 72 °C for 1.5 min, and (c) a final extension step at 72 °C for 7 min. Unincorporated dNTP, and primers were removed using Corning 384 FiltrEX plates (Corning, Cat No 3533), according to the manufacturer’s instructions. Sequencing reactions were set using 2 μL of the cleaned PCR products and the P9 primer (5’ gga att cta tcc tac tat gag aaa aat gag g 3’) with the BigDye terminator v3.1 Cycle sequencing kit (ABI, Cat No 4337456), according to the manufacturer’s instructions. Unincorporated ddNTP were removed with the XTerminator kit (ABI, Cat No 4376484), and samples were analyzed on an ABI 3730 Genetic Analyzer. Raw sequencing data were processed with the Variant Reporter Software v.1.0 (Applied Biosystems). For haplotype determination selected double heterozygous samples were cloned through TA cloning in the pDrive vector (Qiagen, Cat No 231124) and at least ten independent clones were sequenced in both directions.

*PRNP* genotyping data revealed a high degree of variability, with fifteen non-synonymous polymorphisms detected at fourteen codons. Twelve of these were polymorphisms previously observed in Greek goats: *T110P*, *G127S*, *I142M*, *H143R*, *N146S*, R151H, *R154H*, *P168Q*, *S173N*, *R211Q*, *Q222K*, and *S240P*[3,15,16]. Three known polymorphisms were detected for the first time in Greek goats (*W102G*, *N146D* and *I218L*). Of these, the *N146D* dimorphism was found in 4 heterozygous animals from herds located at different geographical regions (frequency: 0.46%). Similarly, the *I218L* polymorphism was detected in one heterozygous animal. The *W102G* change (frequency 2.18%) was found in tandem with five octarepeats. Four known silent changes were detected at codons 42 (*g* → *a*) (frequency 37.8%), 138 (*t* → *c*) (frequency 37.5%), 179 (*g* → *t*) (frequency 0.1%), and 202 (*c* → *t*) (frequency 0.1%). The codon 179 synonymous change, reported here for the first time in Greek goats, was found in one heterozygous buck in this study. A total of 17 haplotypes were determined (Table 1). The most frequent haplotype included the presence of Proline at codon 240.

**Table 1:** **Haplotype distribution in the Greek goats tested (872 chromosomes examined) **^**1**^

	***PRNP *** **codon number**															
**Haplotype**	**102**	**110**	**127**	**142**	**143**	**146**	**151**	**154**	**168**	**173**	**211**	**218**	**222**	**240**	**Haplotype No.**	**Frequency %**
*S*_*240*_														*S*	147	16.9
*G*_*102*_*S*_*240*_	*G*													*S*	19	2.2
*P*_*110*_*S*_*240*_		*P*												*S*	37	4.2
*S*_*127*_*P*_*240*_			*S*											*P*	19	2.2
*M*_*142*_*P*_*240*_				*M*										*P*	13	1.5
*R*_*143*_*P*_*240*_					*R*									*P*	4	0.5
*D*_*146*_*P*_*240*_						*D*								*P*	4	0.5
*S*_*146*_*P*_*240*_						*S*								*P*	26	3.0
*H*_*151*_*S*_*240*_							*H*							*S*	1	0.1
*H*_*154*_*P*_*240*_								*H*						*P*	3	0.3
*H*_*154*_*S*_*240*_								*H*						*S*	13	1.5
*Q*_*168*_*P*_*240*_									*Q*					*P*	15	1.7
*N*_*173*_*P*_*240*_										*N*				*P*	2	0.2
*Q*_*211*_*S*_*240*_											*Q*			*S*	52	6.0
*L*_*218*_*S*_*240*_												*L*		*S*	1	0.1
*K*_*222*_*S*_*240*_													*K*	*S*	49	5.6
*P*_*240*_														*P*	467	53.6
														Total	872	100.0

^1^The *PRNP* coding region of 436 healthy Greek goats included in this study was analyzed by DNA sequencing, as described in the main text. Haplotypes were determined through TA cloning of double heterozygous samples. Haplotype frequencies in the tested population are shown.

The analysis of a cohort of 187 healthy bucks from 38 herds, revealed relatively high frequencies of the scrapie-protective *Q211* (6.7%), *K222* (3.7%), and *S146* (3.5%) alleles. Among other detected alleles, the moderately protective *H154* and *R143* polymorphisms occurred at lower frequencies (1.9% and 0.5% respectively). Thirty-four different genotypes were associated with the male samples analyzed. Interestingly, almost half of these genotypes, 16, were found to harbour one of the alleles associated with scrapie-resistance (*M142*, *R143*, *N146*, *H154*, *Q211*, *K222*), accounting for 30.5% of the tested males. Among these, *Q211*-associated genotypes were the most abundant (5 genotypes, 11.2% of tested bucks), followed by those bearing the *N146* (4 genotypes, 7% of tested males) and *K222* alleles (3 genotypes, 5.9%). Individuals carrying two scrapie-resistance associated alleles, including one *K222* and one *Q211* homozygote, were also detected, but at a much lower frequency (2.1%). This information is provided in more detail in an Additional file (see Additional file 1).

## Discussion

In the present study we analyzed a large number of healthy goats from different holdings, in order to characterize the *PRNP* variability in the Greek goat population. As expected, the caprine *PRNP* gene displayed a high degree of variability. The non-synonymous polymorphisms found included twelve previously reported changes [3,15,16] and three that were novel for the Greek goat population. Of the newly detected alleles, the *N146D* polymorphism, which has also been found in Cypriot goats [4], is strongly associated with resistance against scrapie. The *I218L* dimorphism has been reported in both Chinese [18] and British goats [19]. Finally, the *W102G* dimorphism was originally observed in tandem with a reduced number of octapeptide repeats (three instead of five) [20]. In our study, however, this allele was found in tandem with the wild type five octarepeats, as reported by other recent studies [4,21,22]. Four known silent changes were detected in the tested population. One of these, the codon 179 synonymous change, is reported for the first time in Greek goats, but has previously been found in Cypriot [22], and Spanish goats [23].

In contrast to studies on goat populations from Northern Europe, but consistent with previous studies of Greek, Cypriot [22], and Italian [24] goat populations, *P240* was found to be the most abundant *PRNP* allele. As in other countries, the *Q211* and *K222* alleles were found in linkage with the *S240* allele, and they were also frequently linked with the minor alleles of silent changes at codons 42 (*a*) and 138 (*c*). Although silent changes do not affect the PrP amino acid composition, their importance should not be discounted, since they may be linked with RNA processing or with genetic loci associated with important production traits. Indeed, a recent publication on Chinese goats [25] reported the linkage of codon 42 with milk traits in some goat breeds.

Interestingly, the *Q211*, *S146* and *K222 PRNP* alleles, for which a strong protective effect against goat scrapie was suggested by field [4-7] and in vitro studies [14], and further confirmed by recently published studies on experimentally challenged goats [8-10] and on transgenic mice [11], were detected in relatively high frequencies in the tested population. This result depicts the special situation of Greek goats with respect to the frequency of the before mentioned alleles, as they represent the only goat population among European countries where all three most scrapie-protective alleles are detected. Indeed, even though the *Q211* and *K222* alleles have been found in relatively high frequencies in some countries [6,24], the *S146* allele was absent from these, and other populations [23], while in other cases the *K22*2 allele was represented in very low frequencies [23,19].

Even though goat scrapie prevalence is high in Greece, a clear association between specific caprine *PRNP* alleles and scrapie protection has not been determined, due to the lack of large-scale case-control studies. Nevertheless, a meta-analysis performed on the published data referring to *PRNP* genotypes from scrapie-affected Greek goats may lead to quite strong estimations. The analysis included 146 scrapie-positive cases from 36 herds [3,15,16], where the following allelic frequencies were detected: *S49 (0.3%)*, *P110 (1%)*, *S127 (0.7%)*, *T136 (0.3%)*, *M142 (0.7%)*, *R143 (0.7%), H151 (0.3%)*, *H154 (2.4%), Q154 (0.3%)*, *Q168 (0.7%)*, and *K222 (1.7%)*[3,15,16]. Importantly, no goat scrapie-cases have been reported for *Q211* or *S146* carriers, suggesting that the probabilities of disease occurrence in these animals are low.

The detection of *K222* carriers within scrapie affected animals, which has also been reported in French goats [6], verifies that this allele does not confer complete resistance to goat-scrapie. It should, however, be emphasized that *K222* was found in five heterozygous scrapie-positive animals, originating from three heavily affected herds, associated with a scrapie-isolate displaying a distinct molecular profile as compared to other Greek isolates [16]. The same allele was detected in a much higher frequency (4.2%) in healthy animals (1222 animals, corresponding to relevant data from all three previously published studies and the study presented here). Even though these data do not correspond to a case-study, they still provide valuable information on the protective effect of the *K222* allele. This claim is also strengthened by the fact that no scrapie cases were detected in *K222* homozygous goats, even though the latter observation is only an indication that cannot be verified due to the low number of *K222* homozygous animals. Taken together, the above data suggest that (a) the *K222* allele displays a scrapie-protective effect in Greek goats, and that (b) a herd’s scrapie history, as well as the scrapie strain, are important factors that should be considered before selecting the most appropriate *PRNP* alleles for scrapie-resistance breeding.

Based on the previous data and on the results from experimental challenges in goats [8-10] and transgenic mice [11], it seems that *Q211, S146 and K222* alleles are potential candidates for selective goat-scrapie resistance breeding programs in Greece, provided that herd history and scrapie strain information are also available. Such programs should be most efficient when bucks carrying the desired alleles are recruited. This is because in the Greek farming system the male to female ratio in a holding is usually 1:20. Thus, bucks have a strong impact on the determination of the overall genetic profile of the herd. Our results indicate that the *Q211*, *S146,* and *K222* alleles are present in relatively high frequencies in both the general population, and in bucks specifically, suggesting that a scheme targeting the selection of bucks carrying these alleles is feasible. In contrast, other resistance-associated alleles (*M142*, *R143* and *H154*) could not be targeted, due to their much lower frequencies in bucks. Furthermore, the *H154* allele has been associated with increased susceptibility to atypical scrapie in both sheep and goats [26,27]. Thus, the feasibility of incorporating *M142*, *R143*, and *H154* alleles into a selective breeding scheme is low.

Breeding for resistance in goats by recruiting bucks harbouring the *Q211*, *S146*, and *K222* alleles, should result in a rapid increase of their frequencies in the overall population, which would be expected to reduce the individuals’ risk for developing scrapie. The application of such a strategy would be expected to effect a quick halt on the disease spread within a scrapie-affected herd, and to reduce a herd’s overall risk of becoming scrapie-affected, in a scrapie-free herd. Indeed, available data on scrapie-affected flocks of sheep show that breeding for scrapie resistance with *ARR/ARR* rams results in rapid control of scrapie-outbreaks [28]. Furthermore, it has been shown that a flock’s *PRNP* profile is a risk factor for scrapie occurrence [29]. Increased frequencies of the resistance-associated *ARR* allele, as a result of selective breeding, have a population effect, reducing the scrapie infection risk even for animals of susceptible genotypes [30].

Such an aggressive scheme as the one described above for sheep, cannot be directly applied to Greek goat populations, because (a) there seems to be more than one allele associated with high -but not absolute- scrapie resistance, and (b) the frequency of bucks homozygous for either the *Q211* and/or *K222* allele is low, and no males homozygous for the *S146* allele have been detected. Given that the observed frequencies of the *Q211*, *S146*, and *K222* alleles are moderate to high, a selection scheme targeting carriers of all three of these alleles seems to be more feasible. Furthermore, this strategy conserves more of the original variability of the population. Variability is desired both with respect to protection against other scrapie strains affecting the resistance-associated alleles, as well as with respect to preservation of production traits possibly related to *PRNP*. Breeding for a homogenous population poses a risk for the emergence of an uncontrolled epidemic in the event that a strain targeting the previously resistant genotypes should predominate.

A selective breeding scheme aiming at the increase of the protective *Q221*, *S146* and *K222* alleles is expected to decrease the goat-scrapie incidence and to significantly contribute to the control of scrapie in small ruminants. This is because scrapie affected goats may serve as reservoirs for the disease agent, which can subsequently be transmitted to sheep. The possibility of this transmission to occur is thought to be high when taking into consideration that (a) the main farming system in Greece includes mixed sheep and goat flocks and that (b) the disease occurs more often in sheep than in goats [3]. Thus, the reduction of scrapie incidence in goats, in combination with scrapie reduction in sheep due to the application of the corresponding sheep-scrapie eradication program, is expected to strengthen the result of the latter and to have a beneficial effect on the control of the disease in small ruminants in general. This is highly relevant not only for Greece, but also for other countries where sheep scrapie resistance programs are applied.

Further, the control of scrapie is also expected to improve animal welfare and to highly benefit stakeholders, by reducing costs due to animal loss and by increasing sheep and goat farming income and the dairy products related to the agricultural sector, which significantly contributes to the total agricultural income.

In summary, the present study is intended to extend current knowledge on *PRNP* genetic variability in the Greek goat population, one of the biggest in Europe, and to provide valuable information on scrapie resistance-associated allele frequencies found in male-goats. Further, based on the results presented here and on other studies on Greek goat *PRNP* alleles and their association with scrapie, we suggest the design of a goat-scrapie resistance program targeting the *Q211*, *S146* and *K222* alleles. The implementation of such a program is expected, to our opinion, to greatly aid the control of scrapie in small ruminants and to highly benefit the relevant stakeholders in Greece. Taking into account the special situation in Greece, where one of the largest goat populations in Europe resides and all three most protective goat-scrapie alleles are detected in relatively high frequencies in both the general population and in bucks, it follows that the implementation of the breeding program proposed here, seems feasible. Moreover, the application of the suggested scheme in Greek goats would serve as an example of the results such a strategy would have on the control of the disease in small ruminants and of its beneficial effects on relative stakeholders in general.

## Abbreviations

PRNP: Prion protein encoding gene; SNPs: Single Nucleotide Polymorphisms; PrPC: normal prion protein; PrPSc: Rich in β-sheet prion protein isoform.

## Competing interests

The authors declare that they have no competing interests.

## Authors’ contributions

TS, EK conceived the study and participated in its design. NG performed the sampling, EK performed the genetic analysis. CP, EK carried out the data analysis and participated in the draft manuscript preparation. TS, MA, NP reviewed the final manuscript. All authors read and approved the final manuscript.

## Supplementary Material

Additional file 1 **Genotypes detected in the 187 healthy Greek bucks analyzed in this study.** The *PRNP* coding region of 187 healthy Greek bucks included in this study was analyzed by DNA sequencing, as described in the main text. Haplotypes were determined through TA cloning of double heterozygous samples and subsequent sequencing of ten independent clones in both directions. Genotypes were assigned based on the determined haplotypes. Click here for file
