# Supplementary material for: Perspectives of a scrapie resistance breeding scheme targeting Q211, S146 and K222 caprine PRNP alleles in Greek goats
Source: Vet Res. 2014 Apr 9;45(1):43. doi: 10.1186/1297-9716-45-43 (PMC4030296; doi:10.1186/1297-9716-45-43)
Supplement: Additional file 1 — Genotypes detected in the 187 healthy Greek bucks analyzed in this study. The PRNP coding region of 187 healthy Greek bucks included in this study was analyzed by DNA sequencing, as described in the main text. Haplotypes were determined through TA cloning of double heterozygous samples and subsequent sequencing of ten independent clones in both directions. Genotypes were assigned based on the determined haplotypes. [file 1297-9716-45-43-S1.doc]

**Additional file 1 Genotypes detected in the 187 healthy Greek bucks analyzed in this study ^1^**.

|  | *PRNP* codon number | | | | | | | | | | | | |  |  |
| --- | --- | --- | --- | --- | --- | --- | --- | --- | --- | --- | --- | --- | --- | --- | --- |
| Genotype | 102 | 110 | 127 | 142 | 143 | 146 | 154 | 168 | 173 | 211 | 218 | 222 | 240 | Animal No | Total % |
| *^#^IM_142_PP_240_* |  |  |  | *IM* |  |  |  |  |  |  |  |  | *PP* | 3 | 1.6 |
| *^#^HR_143_PP_240_* |  |  |  |  | *HR* |  |  |  |  |  |  |  | *PP* | 2 | 1.1 |
| *^#^NS_146_PP_240_* |  |  |  |  |  | *NS* |  |  |  |  |  |  | *PP* | 6 | 3.2 |
| *^#^NS_146_PS_240_* |  |  |  |  |  | *NS* |  |  |  |  |  |  | *PS* | 5 | 2.7 |
| *^#^WG_102_NS_146_PS_240_* | *WG* |  |  |  |  | *NS* |  |  |  |  |  |  | *PS* | 1 | 0.5 |
| *^#^NS_146_SN_173_PP_240_* |  |  |  |  |  | *NS* |  |  | *SN* |  |  |  | *PP* | 1 | 0.5 |
| *^#^RH_154_PS_240_* |  |  |  |  |  |  | *RH* |  |  |  |  |  | *PS* | 5 | 2.7 |
| *^#^RH_154_SS_240_* |  |  |  |  |  |  | *RH* |  |  |  |  |  | *SS* | 2 | 1.1 |
| *^#^RQ_211_PS_240_* |  |  |  |  |  |  |  |  |  | *RQ* |  |  | *PS* | 16 | 8.6 |
| *^#^RQ_211_SS_240_* |  |  |  |  |  |  |  |  |  | *RQ* |  |  | *SS* | 2 | 1.1 |
| *^#^WG_102_RQ_211_SS_240_* | *WG* |  |  |  |  |  |  |  |  | *RQ* |  |  | *SS* | 1 | 0.5 |
| *^#^PQ_168_RQ_211_PS_240_* |  |  |  |  |  |  |  | *PQ* |  | *RQ* |  |  | *PS* | 1 | 0.5 |
| *^#^RQ_211_IL_218_SS_240_* |  |  |  |  |  |  |  |  |  | *RQ* | *IL* |  | *SS* | 1 | 0.5 |
| *^#^QK_222_PS_240_* |  |  |  |  |  |  |  |  |  |  |  | *QK* | *PS* | 9 | 4.8 |
| *^#^QK_222_SS_240_* |  |  |  |  |  |  |  |  |  |  |  | *QK* | *SS* | 1 | 0.5 |
| *^#^GS_127_QK_222_PS_240_* |  |  | *GS* |  |  |  |  |  |  |  |  | *QK* | *PS* | 1 | 0.5 |
| *^#^*Total (1) |  |  |  |  |  |  |  |  |  |  |  |  |  | 57 | 30.5 |
| *^§^IM_142_RQ_211_PS_240_* |  |  |  | *IM* |  |  |  |  |  | *RQ* |  |  | *PS* | 1 | 0.5 |
| *^§^QQ_211_SS_240_* |  |  |  |  |  |  |  |  |  | *QQ* |  |  | *SS* | 1 | 0.5 |
| *^§^RQ_211_QK_222_SS_240_* |  |  |  |  |  |  |  |  |  | *RQ* |  | *QK* | *SS* | 1 | 0.5 |
| *^§^KK_222_SS_240_* |  |  |  |  |  |  |  |  |  |  |  | *KK* | *SS* | 1 | 0.5 |
| *^§^*Total (2) |  |  |  |  |  |  |  |  |  |  |  |  |  | 4 | 2.1 |
| *±GG_102_SS_240_* | *GG* |  |  |  |  |  |  |  |  |  |  |  | *SS* | 1 | 0.5 |
| *±WG_102_PS_240_* | *WG* |  |  |  |  |  |  |  |  |  |  |  | *PS* | 3 | 1.6 |
| *±WG_102_TP_110_SS_240_* | *WG* | *TP* |  |  |  |  |  |  |  |  |  |  | *SS* | 3 | 1.6 |
| *±TP_110_PS_240_* |  | *TP* |  |  |  |  |  |  |  |  |  |  | *PS* | 6 | 3.2 |
| *±TP_110_SS_240_* |  | *TP* |  |  |  |  |  |  |  |  |  |  | *SS* | 2 | 1.1 |
| *±TP_110_PQ_168_PS_240_* |  | *TP* |  |  |  |  |  | *PQ* |  |  |  |  | *PS* | 1 | 0.5 |
| *±GS_127_PP_240_* |  |  | *GS* |  |  |  |  |  |  |  |  |  | *PP* | 3 | 1.6 |
| *±SS_127_PP_240_* |  |  | *SS* |  |  |  |  |  |  |  |  |  | *PP* | 2 | 1.1 |
| *±PQ_168_PP_240_* |  |  |  |  |  |  |  | *PQ* |  |  |  |  | *PP* | 3 | 1.6 |
| *±PQ_168_PS_240_* |  |  |  |  |  |  |  | *PQ* |  |  |  |  | *PS* | 4 | 2.1 |
| *±SN_173_PP_240_* |  |  |  |  |  |  |  |  | *SN* |  |  |  | *PP* | 1 | 0.5 |
| *±PP_240_* |  |  |  |  |  |  |  |  |  |  |  |  | *PP* | 47 | 25.1 |
| *±PS_240_* |  |  |  |  |  |  |  |  |  |  |  |  | *PS* | 48 | 25.7 |
| *±SS_240_* |  |  |  |  |  |  |  |  |  |  |  |  | *SS* | 2 | 1.1 |
| *±*Total (None) |  |  |  |  |  |  |  |  |  |  |  |  |  | 126 | 67.4 |
| Total |  |  |  |  |  |  |  |  |  |  |  |  |  | 187 | 100.0 |

^1^ **^#^** Genotypes bearing one scrapie-resistance associated allele (1), **^§^** Genotypes bearing two scrapie-resistance associated alleles (2), **±** Genotypes with no scrapie-resistance associated alleles (None).
